# The Positive Impact of Community-Based Health Support on the Utilization of Preventive Healthcare Among Older Adults: An Analysis of Cross-Sectional Data From China

**DOI:** 10.7759/cureus.85157

**Published:** 2025-05-31

**Authors:** Huan Song, Hui Sun

**Affiliations:** 1 School of Public Administration, Nanjing Normal University, Nanjing, CHN; 2 College of Media and Art, Nanjing University of Posts and Telecommunications, Nanjing, CHN

**Keywords:** china, community-based health support, older adults, physical examinations, preventive healthcare

## Abstract

Aim: This study aimed to analyze the role of service support from community health centers in promoting the utilization of preventive physical examinations among older adults.

Methods: Cross-sectional data from 6461 respondents were obtained from the 2018 China Health and Retirement Longitudinal Study (CHARLS). Community-based health support was assessed based on whether community health centers provided physical examinations for older adults. The utilization of preventive healthcare for older adults was measured by their participation in physical examinations. A logistic regression model, propensity score matching (PSM), and doubly robust estimation were employed.

Results: The sample characteristics analysis presented that the majority of older adults in this study did not receive preventive physical examination services from community health centers. After adjusting for control variables and conducting robustness tests, the results showed that community-based health support is a significant and robust positive factor in enhancing the possibility of preventive healthcare utilization among Chinese older adults. Moreover, the magnitude of the association varies with the age, education, disability, and area and region of residence of older adults.

Conclusion: The findings underscored the beneficial role of community-based health support in promoting the utilization of preventive healthcare among older adults. Healthcare policymakers should prioritize community-centered policies to promote preventive health behaviors among older adults.

## Introduction

China is currently experiencing a rapid process of population aging, with the percentage of its population aged 65 and older projected to double from 14.2% in 2021 to 26.9% in 2050 [1,2]. The aging population has raised concerns about the vulnerability of health and the utilization of preventive healthcare in later life. Several studies have measured the utilization of preventive healthcare by examining participation in physical examinations and emphasized the crucial role of prevention in maintaining the health of older adults [3,4]. Preventive healthcare is often considered more discretionary or optional for individuals than curative healthcare [5]. Analysis of qualitative data has revealed a widespread lack of consensus on the components of preventive healthcare and showed that most older adults define prevention as a healthy diet and exercise routine rather than screening for disease or necessary clinical services [6]. The healthcare system in China has limited capacity to provide preventive healthcare to all citizens [7]. It is essential to understand the factors that promote the utilization of preventive healthcare among older adults to maximize the effectiveness of limited healthcare resources.

Numerous studies have identified various factors that impact healthcare utilization among older adults, including geographic location, health needs, and economic status [8-10]. Some scholars have attempted to explore the factors that influence the utilization of preventive healthcare from a similar perspective. For instance, Chen et al. proposed that health literacy, as assessed by educational level, cognitive ability, and knowledge of diseases, affects the utilization of preventive healthcare among older adults [11]. Yamaguchi et al. analyzed the characteristics of older adults who participated in a community physical examination program in Japan, including their family structure, self-reported health, and physical function [12]. The study conducted by Fu et al. revealed that in China, socioeconomic and educational factors have a strong influence on the utilization of preventive healthcare among older adults and that utilization varied across groups [13]. Studies have also examined the effect of older adults’ religious beliefs and trust in the healthcare system [14,15].

An increasing number of recent studies have focused on the positive effects of community-based healthcare services on the health of older adults, particularly those with disabilities [16-18]. Several studies have analyzed the demand for and supply of various types of community-based healthcare services for older adults in China, including personal care, home visits, psychological counseling, medical support, and health education [19,20]. Yu et al. emphasized the significance of community-based preventive healthcare services in improving the overall health of the population in their policy discussion aimed at developing community-based healthcare in Shanghai, China [21]. A study was conducted in the United States to analyze the effect of community-based physician availability on the utilization of preventive healthcare [22]. Community-based healthcare is the foundation for disease prevention and management. Relatively few studies have been conducted on community-based preventive healthcare for older adults in China.

This study aimed to investigate the correlation between community-based health support and the utilization of preventive healthcare among Chinese older adults and to analyze how this relationship varies across different groups of older adults. Community-based health support was measured by assessing whether community health centers provided physical examination services for older adults. The participation of older adults in physical examinations was used to measure the utilization of preventive healthcare. By employing a cross-sectional design and nationally representative data, this study hypothesized that community-based health support has a positive effect on promoting the utilization of preventive healthcare among older adults. This effect was hypothesized to vary based on the demographic characteristics, physical functioning, and place of residence. The results emphasized the significant role that community-based health support plays in promoting the utilization of preventive healthcare among older adults and served as a reference for formulating relevant policies.

## Materials and methods

Data and sample

This study used publicly available cross-sectional data from the 2018 China Health and Retirement Longitudinal Study (CHARLS). The CHARLS is a nationally representative longitudinal survey and covers 28 provinces, municipalities, and autonomous regions in China. Its response rate and credibility were both high [23,24]. The CHARLS was approved by the Peking University Biomedical Ethics Committee (Approval No.: IRB00001052-11015) and obtained the written informed consent of all participants. A total of 19,816 respondents participated in the 2018 CHARLS. The 2018 CHARLS data provides extensive information on demographic characteristics, living arrangements, economic resources, behavioral activities, physical and mental health, as well as healthcare needs and provisions.

As this study aimed to investigate the utilization of preventive healthcare and community-based healthcare support among older adults, the research subjects were required to meet the following inclusion criteria: (a) should be 60 years or older and (b) have complete information available on physical examinations and community-based healthcare. Furthermore, this study gathered data on regional healthcare resources, including the number of healthcare beds and technicians per 10,000 residents, from the 2018 China Statistical Yearbook. This information was then merged with the 2018 CHARLS dataset. After removing missing values from the other control variables, the final sample size for the analysis was 6461.

Dependent variable

The dependent variable in this study was the utilization of preventive healthcare. Physical examinations are a primary form of preventive healthcare. This study defined preventive healthcare utilization among older adults as their participation in physical examinations. Participants in the 2018 CHARLS were asked to answer “When did you take the last physical examination.” Respondents who had undergone a physical examination within the past year were classified as having utilized preventive healthcare and were assigned a code of 1; otherwise, they were assigned a code of 0. Accordingly, the utilization of preventive healthcare was described as a dichotomous variable. The reference group consisted of older adults who had not sought preventive healthcare in the past year.

Independent variable

The independent variable in this study was community-based health support, which was dichotomized into two categories: 1 for “yes” and 0 for “no.” The 2018 CHARLS asked respondents to select from several items which healthcare services the community health center provided for them, including regular physical examinations. If the respondent selected regular physical examination, it was considered that the community provided preventive healthcare support for older adults and could be assigned a code of 1. Otherwise, it was assigned a code of 0.

Control variables

This study used the Andersen’s Behavioral Model of Health Service Utilization to define the dimensional framework of control variables, which included predisposing, enabling, and need factors. This approach was adopted to better control for other confounding variables that may influence the utilization of preventive healthcare among older adults. Numerous studies have employed Andersen’s model to examine the individual and contextual factors that influence healthcare utilization [25,26]. Some studies have indicated that economic and social support are important enabling resources [27,28]. This study categorized the enabling factors into individual socioeconomic status and social support networks. Disparities in personal healthcare utilization can be attributed to physical conditions and psychological factors [12,29]. The need factors in this study were described as both physical and psychological. This study also included lifestyle factors as control variables, in accordance with previous studies [8]. The measurements of the control variables are shown in Table 1.

**Table 1:** Measurements of control variables Note: Eastern regions of China: Shanghai, Beijing, Tianjin, Shandong, Guangdong, Jiangsu, Hebei, Zhejiang, Fujian, and Liaoning. Central regions of China: Jilin, Anhui, Jiangxi, Henan, Hubei, Shanxi, Hunan, and Heilongjiang. Western regions of China: Yunnan, Sichuan, Guangxi, Chongqing, Gansu, Xinjiang, Guizhou, Qinghai, Inner Mongolia, and Shaanxi. ADL: activities of daily living, IADL: instrumental activities of daily living.

Variable	Measurements
Predisposing factors	
Age (in years)	A continuous variable
Gender	1 = female, 0 = male
Marital status	2 = married, 1 = widowed, 0 = divorced/never married
Ethnicity	1 = Han, 0 = Minority
Area	1 = living in urban areas, 0 = living in rural areas
Region	2 = living in the Eastern region, 1 = living in the Central region, 0 = living in the Western region
Enabling factors	
Socioeconomic status	
Education	1 = literate, 0 = illiterate
Health insurance	1 = yes, 0 = no
Disposable cash	A continuous variable
Annual healthcare expenditure	A continuous variable
Social support networks	
Social participation	1 = yes, 0 = no
Local healthcare services satisfaction	4 = very high, 3 = high, 2 = mediate, 1 = low, 0 = very low
Number of healthcare beds per 10,000 residents	A continuous variable
Number of healthcare technicians per 10,000 residents	A continuous variable
Need factors	
Physical needs	
Disability	1 = ADL or IADL disability, 0 = none
Number of chronic diseases	A continuous variable
Cognitive ability scores	A continuous variable; a higher score means a better cognitive state
Psychological needs	
Self-reported health	2 = good, 1 = fair, 0 = poor
Life satisfaction	4 = very high, 3 = high, 2 = mediate, 1 = low, 0 = very low
Depressive symptom scores	A continuous variable; a higher score means a more severe depressive state
Lifestyle factors	
Smoking	2 = current smoker, 1 = former smoker, 0 = never smoked
Drinking	2 = more than once a month, 1 = less than once a month, 0 = never drank
Doing exercise	1 = yes, 0 = no

Statistical analysis

The mean, standard deviation (SD), maximum, and minimum values were used to describe the basic characteristics of the study sample. The logistic regression model was employed to analyze the association of community-based health support with the utilization of preventive healthcare among older adults. Propensity score matching (PSM) is based on a causal counterfactual framework and can make observational data closely resemble randomized experimental data. As a result, many studies have used PSM to address selection bias [30,31]. In this study, the PSM method was used for robustness testing, including k-nearest neighbor matching, radius matching, kernel matching, and nearest-neighbor matching within a caliper. The doubly robust estimation method combines propensity score weighting with regression models [32,33]. The study’s robustness analysis also introduced two methods for doubly robust estimation: augmented inverse-probability weighting (AIPW) and inverse-probability-weighted regression adjustment (IPWRA). Additionally, this study grouped the sample based on age, education, disability, area, and region to analyze the heterogeneous effects of community-based health support on the utilization of preventive healthcare among various groups of older adults. The data was analyzed using Stata Version 17 (StataCorp LLC, College Station, TX, US). All statistical tests were two-sided, and a p-value of less than 0.05 was considered statistically significant.

## Results

Sample characteristics

Table 2 presents the basic characteristics of the study sample. Out of the 6,461 respondents analyzed in this study, the majority reported that they did not receive preventive physical examination services from community health centers. Moreover, most of the interviewed older adults (a) lived in rural areas and Central-Eastern regions; (b) were educated, had health insurance, but expressed dissatisfaction with local healthcare services; (c) had disabilities, at least one chronic disease, cognitive impairment, poor self-reported health, and low life satisfaction; and (d) engaged in less daily exercise. The large SDs of disposable cash, annual healthcare expenditures, and number of healthcare beds and technicians indicated significant disparities in financial and healthcare resources among the surveyed older adults.

**Table 2:** Characteristics of the study sample (N = 6461)

Variable	Mean	SD	Min	Max
Physical examination	0.534	0.499	0	1
Community-based health support	0.172	0.378	0	1
Predisposing factors				
Age (in years)	68.566	6.489	60	108
Gender	0.504	0.500	0	1
Marital status	1.802	0.430	0	2
Ethnicity	0.943	0.232	0	1
Area	0.274	0.446	0	1
Region	1.140	0.768	0	2
Enabling factors				
Socioeconomic status				
Education	0.721	0.448	0	1
Health insurance	0.974	0.158	0	1
Disposable cash	2143.747	77,27.803	0	400,000
Annual healthcare expenditure	6596.182	16,445.084	0	360,000
Social support networks				
Social participation	0.507	0.500	0	1
Local healthcare service satisfaction	2.336	1.139	0	4
Number of healthcare beds per 10,000 residents	58.425	25.159	34	121
Number of healthcare technicians per 10,000 residents	65.563	31.409	34	139
Need factors				
Physical needs				
Disability	0.594	0.491	0	1
Number of chronic diseases	0.787	1.087	0	11
Cognitive ability scores	7.289	3.634	0	18
Psychological needs				
Self-reported health	0.944	0.717	0	2
Life satisfaction	2.297	0.802	0	4
Depressive symptom scores	7.300	6.120	0	28
Lifestyle factors				
Smoking	0.723	0.859	0	2
Drinking	0.579	0.867	0	2
Doing exercise	0.408	0.492	0	1

Regression analysis

Table 3 reports the regression results of the relationship between community-based health support and the utilization of preventive healthcare among older adults. The results of Model 1 indicated that community-based health support significantly increased the likelihood of utilizing preventive healthcare, with an odds ratio of 7.709 (p < 0.001). As control variables were gradually included in Models 2-5, the odds ratio values decreased, while the significance level remained unchanged. Based on Model 1, Model 2 incorporated predisposing factors with an odds ratio of 7.194 (p < 0.001), and Models 3 and 4 included enabling and need factors with odds ratios of 6.805 (p < 0.001) and 6.623 (p < 0.001), respectively. Model 5 added lifestyle factors with an odds ratio of 6.588 (p < 0.001). The regression results suggested that community-based health support was a significant positive factor in increasing the possibility of the utilization of preventive healthcare among older adults.

According to Model 5, it can be inferred that several factors have a significant positive effect on the likelihood of older adults utilizing preventive healthcare. These factors include age, ethnicity, disposable cash, healthcare expenditure, social participation, satisfaction with healthcare services, number of healthcare technicians, chronic diseases, cognitive ability, and regular exercise. However, the number of healthcare beds, self-reported health, and smoking are negative factors that decrease the likelihood of utilizing preventive healthcare. Specifically, older adults who were of Han Chinese ethnicity, socially engaged, and engaged in exercise, as well as those with higher disposable income, greater annual healthcare expenditures, higher satisfaction with local healthcare services, better cognitive ability, and a greater number of chronic diseases and healthcare technicians in their area of residence, were more likely to utilize preventive healthcare. Older adults who were current smokers, resided in areas with more healthcare beds, and reported better health were less likely to utilize preventive healthcare.

**Table 3:** Odds ratio of preventive healthcare utilization among older adults Note: Robust standard errors were reported in parentheses. ^+^p < 0.1, *p < 0.05, **p < 0.01, ***p < 0.001.

Variable	Model 1	Model 2	Model 3	Model 4	Model 5	
	(N = 6461)	(N = 6461)	(N = 6461)	(N = 6461)	(N = 6461)	
Community-based health support	7.709***	7.194***	6.805***	6.623***	6.588***	
	(0.718)	(0.675)	(0.651)	(0.640)	(0.642)	
Predisposing factors						
Age (in years)		1.038***	1.043***	1.050***	1.047***	
		(0.005)	(0.005)	(0.005)	(0.005)	
Gender		1.114*	1.187**	1.207**	1.025	
		(0.060)	(0.070)	(0.073)	(0.081)	
Marital status		1.212**	1.124^+^	1.078	1.073	
		(0.081)	(0.078)	(0.076)	(0.076)	
Ethnicity		1.454**	1.514***	1.452**	1.443**	
		(0.174)	(0.183)	(0.180)	(0.180)	
Area		1.294***	0.974	0.876	0.815	
		(0.078)	(0.171)	(0.155)	(0.145)	
Region		1.116**	1.001	1.006	1.017	
		(0.040)	(0.038)	(0.039)	(0.040)	
Enabling factors						
Socioeconomic status						
Education			1.228**	0.930	0.924	
			(0.083)	(0.071)	(0.071)	
Health insurance			1.710**	1.616**	1.587*	
			(0.308)	(0.300)	(0.294)	
Log (disposable cash)			1.049***	1.041***	1.040***	
			(0.011)	(0.011)	(0.012)	
Log (annual healthcare expenditure)			1.022***	1.016**	1.015**	
			(0.006)	(0.006)	(0.006)	
Social support networks						
Social participation			1.351***	1.290***	1.276***	
			(0.074)	(0.072)	(0.072)	
Local healthcare service satisfaction			1.105***	1.111***	1.113***	
			(0.027)	(0.028)	(0.028)	
Number of healthcare beds per 10,000 residents			0.968***	0.967***	0.968***	
			(0.003)	(0.003)	(0.003)	
Number of healthcare technicians per 10,000 residents			1.029***	1.029***	1.029***	
			(0.003)	(0.003)	(0.003)	
Need factors						
Physical needs						
Disability				1.063	1.066	
				(0.060)	(0.060)	
Number of chronic diseases				1.183***	1.175***	
				(0.033)	(0.033)	
Cognitive ability scores				1.084***	1.080***	
				(0.011)	(0.011)	
Psychological needs						
Self-reported health				0.918*	0.913*	
				(0.040)	(0.040)	
Life satisfaction				1.071^+^	1.061	
				(0.040)	(0.040)	
Depressive symptom scores				0.990^+^	0.990^+^	
				(0.005)	(0.005)	
Lifestyle factors						
Smoking					0.853***	
					(0.035)	
Drinking					1.021	
					(0.036)	
Doing exercise					1.270***	
					(0.074)	
Wald χ^2^	480.38***	577.66***	726.07***	802.48***	810.59***	
Pseudo R^2^	0.076	0.090	0.115	0.129	0.133	

Robustness testing

Table 4 provides the estimation results of four PSM methods: k-nearest neighbor matching, radius matching, kernel matching, and nearest-neighbor matching within caliper. It showed that community-based health support had a significant positive effect on the utilization of preventive healthcare among older adults. Table 5 demonstrates the results of the doubly robust estimates of AIPW and IPWRA, which also indicated a significant positive effect of community-based health support on the utilization of preventive healthcare among older adults. The results of the robustness testing supported the main finding of this study, and the logistic regression results were highly robust for further research.

**Table 4:** PSM estimation of the effect of community-based health support on the odds ratio of preventive healthcare utilization Note: The “1:4” matching method was applied in the k-nearest neighbor matching; the caliper was set to 0.01 in radius matching and nearest-neighbor matching within a caliper; normal kernel was used for kernel matching. ATT: average treatment effect on the treated, PSM: propensity score matching, SE: robust standard error. *p < 0.05, **p < 0.01, ***p < 0.001.

Method	Sample	Treated	Control	ATT	SE	T statistic
K-nearest neighbor matching	Unmatched	0.8696	0.4638	0.4058	0.0156	25.93
(N = 6427)	Matched	0.8704	0.5333	0.3371	0.0151	22.30***
Radius matching	Unmatched	0.8696	0.4638	0.4058	0.0156	25.93
(N = 6419)	Matched	0.8703	0.5326	0.3376	0.0129	26.18***
Kernel matching	Unmatched	0.8696	0.4638	0.4058	0.0156	25.93
(N = 6427)	Matched	0.8704	0.5076	0.3628	0.0124	29.23***
Nearest-neighbor matching within caliper	Unmatched	0.8696	0.4638	0.4058	0.0156	25.93
(N = 6419)	Matched	0.8703	0.5334	0.3369	0.0151	22.27***

**Table 5:** Doubly robust estimation of the effect of community-based health support on the odds ratio of preventive healthcare utilization Note: Robust standard errors were reported in parentheses. AIPW: augmented inverse-probability weighting, IPWRA: inverse-probability-weighted regression adjustment, ATE: average treatment effect, ATT: average treatment effect on the treated, PO: probability of outcome. *p < 0.05, **p < 0.01, ***p < 0.001.

	AIPW	IPWRA
	(N = 6461)	(N = 6461)
ATE	0.372***	0.371***
	(0.015)	(0.014)
PO mean	0.476***	0.476***
	(0.007)	(0.007)
ATT		0.337***
		(0.013)
PO mean		0.532***
		(0.009)

Heterogeneity presentation

**Figure 1:**
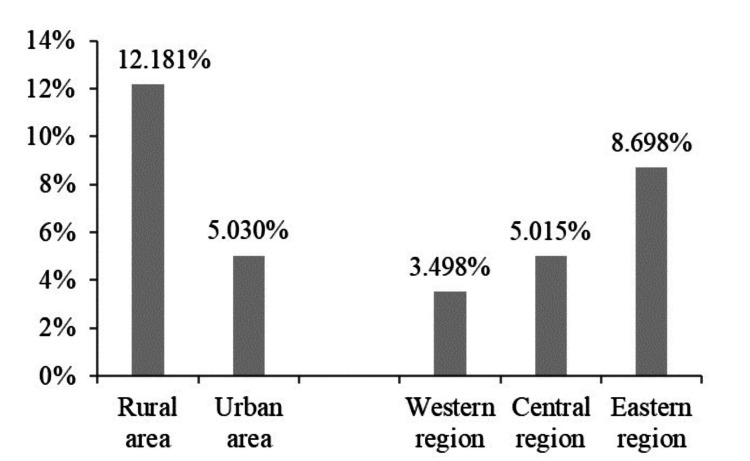
Proportion of community-based health support provided in different areas and regions

The effect of community-based health support on the odds ratio of preventive healthcare utilization among older adults varies with age, education, disability, area, and region, as presented in Table 6. The positive effect of community-based health support on the utilization of preventive healthcare was more pronounced among older adults aged 60-70 years who were illiterate, had ADL or IADL disabilities, and resided in rural areas and Eastern regions. There may be several reasons for this phenomenon, including the inherent heterogeneity in age, education, and physical health among different groups of older adults. Additionally, older adults in rural areas and eastern regions may receive a higher proportion of community-based health support compared to those in urban areas and Central and Western regions, as demonstrated in Figure 1, which is based on data from the current study. In recent years, many health centers in rural communities in China have started offering free physical examinations for older adults, including some routine measurements of height, weight, blood pressure, and blood sugar. It is highly appealing to older adults residing in rural areas who have long endured a shortage of healthcare resources.

**Table 6:** Effects of community-based health support on the odds ratio of preventive healthcare utilization among different groups of older adults Note: Robust standard errors were reported in parentheses. *p < 0.05, **p < 0.01, ***p < 0.001.

Variable	Community-based health support	Control variables	Wald χ^2^	Pseudo R^2^
Age				
60-70	6.791***	Yes	572.81***	0.137
(N = 4334)	(0.837)			
70-80	5.682***	Yes	226.02***	0.137
(N = 1747)	(1.017)			
80+	6.658***	Yes	72.87***	0.184
(N = 380)	(2.666)			
Education				
Literate	5.893***	Yes	646.99***	0.146
(N = 4661)	(0.659)			
Illiterate	9.325***	Yes	208.76***	0.120
(N = 1800)	(1.849)			
Disability				
ADL or IADL disability	7.297***	Yes	452.33***	0.126
(N = 3838)	(0.951)			
None	5.802***	Yes	396.73***	0.154
(N = 2623)	(0.857)			
Area				
Urban	4.496***	Yes	217.07***	0.127
(N = 1769)	(0.790)			
Rural	7.818***	Yes	622.88***	0.139
(N = 4692)	(0.923)			
Region				
East	8.654***	Yes	380.06***	0.179
(N = 2424)	(1.366)			
Central	5.782***	Yes	274.44***	0.110
(N = 2519)	(0.951)			
West	4.803***	Yes	204.51***	0.139
(N = 1518)	(0.967)			

## Discussion

The results of the regression analysis indicated that community-based health support is a significant and robust positive factor in increasing the likelihood of the utilization of preventive healthcare among older adults. It highlighted the crucial role of community support in utilizing preventive healthcare for older adults. Community-based health support has been shown to enhance the accessibility and availability of healthcare for older adults [34-36]. Some studies have emphasized the advantages of community-based healthcare support for the physical and mental well-being of older adults [37,38]. These findings can inspire the development and improvement of relevant policies and measures aimed at enhancing community-based preventive healthcare support for older adults. Previous studies have suggested that community-based healthcare support can be further improved through various means, including increasing government financial support [39], introducing digital technologies [40], and encouraging private sector participation [41].

The analysis of control variables revealed the significance of offering community-based health support for particular priority groups of older adults. The groups include (a) individuals aged 60-70 years with low educational attainment, (b) physically disabled individuals with multiple chronic conditions but higher cognitive ability, (c) those with poor self-reported health status, (d) non-smokers who engage in daily exercise, and (e) individuals residing in rural areas and eastern regions. It is also necessary to enhance economic and social support for older adults, such as providing opportunities for social participation, improving healthcare services to increase their satisfaction, and redirecting the infrastructure’s focus from expanding the number of healthcare beds to technicians. Community-based services for physical examinations of older adults have gradually increased in rural areas and eastern regions due to the support of Chinese central and local government policies in recent years. However, the importance of community-based health support for preventive healthcare for older adults in urban areas, as well as Central and Western regions, cannot be neglected.

The results confirmed that community-based health support can significantly increase the likelihood of older adults utilizing preventive healthcare. Constructing a community-based preventive healthcare program could complement the existing healthcare system for older adults. The study simultaneously analyzed the extent to which the positive relationship between community-based health support and the utilization of preventive healthcare varies among older adults, taking into account factors such as age, education, disability, area, and region. The heterogeneity analysis provided a theoretical reference for the development of more targeted community-based healthcare strategies to promote the utilization of preventive healthcare among different groups of older adults. However, simply increasing the supply of community-based preventive healthcare is not sufficient to ensure that a growing number of older adults adopt preventive health behaviors. Due to the longstanding weakness of primary healthcare in China, residents’ trust in community-based preventive healthcare has been reduced. Policies are also needed to increase older adults’ trust in community-based preventive healthcare. These results also provide a reference for promoting the utilization of preventive healthcare for older adults in other developing countries.

Several limitations of this secondary data analysis have to be mentioned. Firstly, the cross-sectional design of this study means that no causal inferences can be made. Further longitudinal studies are needed to clarify the associations discussed here. Secondly, this study measured the utilization of preventive healthcare among older adults by examining their participation in physical examinations and did not categorize physical examinations based on their content, such as routine examination items and disease-specific screening items. Thirdly, there may be residual confusion and bias introduced by self-reported measurements. Fourthly, due to the inability to obtain the latest relevant data, the results of this study cannot be extended to the impact of the COVID-19 pandemic on the supply and demand of preventive health services. As a result, it failed to provide a more detailed analysis of the relationship between community-based health support and the utilization of preventive healthcare among older adults. Future studies should take these factors into account as more data becomes available.

## Conclusions

Using cross-sectional data from a nationally representative survey, this study found that community-based health support significantly increases the likelihood of older adults utilizing preventive healthcare. Furthermore, the magnitude of this relationship varies with age, education, disability, and area and region of residence of older adults. The findings highlighted the positive role of community-level service support represented by community health centers in promoting the utilization of preventive healthcare among older adults in China. The government should prioritize community-centered policies to promote preventive health behaviors among older adults and accelerate the achievement of healthy aging.
